# Prevalence and orthopedic management of foot and ankle deformities in Charcot–Marie–Tooth disease

**DOI:** 10.1002/mus.25724

**Published:** 2017-07-07

**Authors:** Matilde Laurá, Dishan Singh, Gita Ramdharry, Jasper Morrow, Mariola Skorupinska, Davide Pareyson, Joshua Burns, Richard A. Lewis, Steven S. Scherer, David N. Herrmann, Nicholas Cullen, Christopher Bradish, Luca Gaiani, Nicolò Martinelli, Paul Gibbons, Glenn Pfeffer, Phinit Phisitkul, Keith Wapner, James Sanders, Sam Flemister, Michael E. Shy, Mary M. Reilly, on behalf of the Inherited Neuropathies Consortium

**Affiliations:** ^1^ Medical Research Council Centre for Neuromuscular Diseases University College London Institute of Neurology Queen Square London WC1N 3BG UK; ^2^ Foot and ankle unit Royal National Orthopedic Hospital Stanmore UK; ^3^ School of Rehabilitation Sciences St George's University of London/Kingston University London UK; ^4^ Department of Neurology IRCCS Foundation, Carlo Besta Neurological Institute Milan Italy; ^5^ Faculty of Health Sciences University of Sydney & Children's Hospital at Westmead Sydney New South Wales Australia; ^6^ Department of Neurology Cedars Sinai Medical Center West Hollywood California USA; ^7^ Department of Neurology Hospital of the University of Pennsylvania Philadelphia Pennsylvania USA; ^8^ Department of Neurology University of Rochester Rochester New York; ^9^ Orthopaedic and Spinal Surgery Unit Great Ormond Street Hospital London UK; ^10^ Department of Orthopaedics ASL Imola Italy; ^11^ Department of Ankle and Foot Surgery IRCCS Galeazzi Milan Italy; ^12^ Department of Orthopaedic and Rehabilitation University of Iowa Health Care Iowa City Iowa USA; ^13^ Department of Orthopaedics University of Pennsylvania Philadelphia Pennsylvania USA; ^14^ Department of Orthopaedics University of Rochester Rochester New York USA; ^15^ Department of Neurology University of Iowa health Care, Iowa City Iowa USA

**Keywords:** Charcot–Marie–Tooth disease, foot deformities, foot surgery, orthopedic complications, pes cavus, survey

## Abstract

*Introduction:* Foot deformities are frequent complications in Charcot–Marie–Tooth disease (CMT) patients, often requiring orthopedic surgery. However, there are no prospective, randomized studies on surgical management, and there is variation in the approaches among centers both within and between countries. *Methods:* In this study we assessed the frequency of foot deformities and surgery among patients recruited into the Inherited Neuropathies Consortium (INC). We also designed a survey addressed to orthopedic surgeons at INC centers to determine whether surgical approaches to orthopedic complications in CMT are variable. *Results:* Foot deformities were reported in 71% of CMT patients; 30% of the patients had surgery. Survey questions were answered by 16 surgeons working in different specialized centers. Most of the respondents were foot and ankle surgeons. There was marked variation in surgical management. *Discussion:* Our findings confirm that the approaches to orthopedic management of CMT are varied. We identify areas that require further research. *Muscle Nerve* **57**: 255–259, 2018

Charcot–Marie–Tooth disease (CMT) is the most common inherited peripheral neuropathy.^1^ Clinically, it is characterized by slowly progressive distal wasting, weakness, and sensory loss affecting the lower and upper limbs. Foot deformities, namely forefoot cavus, clawtoes, hindfoot varus, and ankle instability, are frequently observed and are a major source of the disability associated with CMT and commonly result in patients being referred for orthopedic surgery. Muscular imbalance between the weaker tibialis anterior overpowered by the stronger peroneus longus muscles and weakness of intrinsic foot muscles have been suggested as the main causes of forefoot cavus in CMT.^2^, ^3^ Orthopedic surgery is frequently required for CMT patients to correct severe foot deformities. The goals of surgical intervention for patients with CMT are to obtain a plantigrade foot and to correct the bony deformities and muscle imbalance. The indication for surgery is usually determined by failure of conservative measures, such as physical therapy and use of orthoses, which are first‐line treatments for foot deformities in CMT. Several procedures have been described in isolation and in combination for the correction of foot deformities. These include soft tissue procedures, such as plantar fascia release, tendon transfers, and tendon lengthening, which are usually performed for mild flexible deformities. Corrective osteotomies, such as calcaneal osteotomy and first metatarsal osteotomy, may be performed when greater deformity correction is needed or when the deformity has become rigid. Fusion procedures, such as triple arthrodesis, are usually reserved for when there are severe degenerative joint changes and deformities.^4^, ^5^, ^6^ No single surgical procedure can achieve all these goals simultaneously, and surgical decisions are usually individualized.^7^ The long‐term results of surgical procedures on foot deformities in CMT patients have been addressed in just a few studies,^5^, ^6^ so evidence regarding optimal surgical management for these patients is lacking. Furthermore, we have observed that current approaches appear to vary between centers in the same country and between different countries. The aims of this study were to determine: (1) the frequency of foot deformities in CMT and the proportion of patients undergoing foot surgery; and (2) whether current surgical approaches to the management of orthopedic complications in CMT are variable, even in specialized centers.

## METHODS

The study was conducted by the Inherited Neuropathies Consortium (INC), a member of the Rare Diseases Clinical Research Network (RDCRN). The INC is an international consortium performing clinical research and natural history studies in CMT. Ethics approval was obtained by the institutional review board (IRB) at all participating institutions and written informed consent was obtained from all participants.

### INC Patient Foot Deformity Data

Data from patients recruited in the INC Natural History Study of CMT and Related Inherited Neuropathies were interrogated to collect details on diagnosis, presence of foot deformities (pes cavus, hammer toes, pes planus), use of orthotic aids (shoe inserts, ankle foot orthosis), and details of foot surgery (tendon transfer, Achilles tendon lengthening, ankle joint fusion, osteotomy, toe straightening). These data were collected as part of a minimal data set completed by a clinician during assessment for the Natural History Study.^8^

### Survey of Orthopedic Surgeons

A survey was designed by an experienced foot and ankle orthopedic surgeon (D.S.), who specializes in surgery for CMT, and consisted of 6 questions (refer to Supplementary Material, available online). The survey was aimed at experienced CMT orthopedic surgeons and included questions regarding the type of orthopedic practice, number of referrals of patients with severe pes cavus received per year, number of CMT patients seen per year, number of corrections of severe pes cavus performed per year, and number of CMT patients with flat feet encountered. The survey included 2 clinical scenarios of typical CMT patients: a 13‐year‐old and a 24‐year‐old CMT patient with typical pes cavus who had failed conservative measures. The orthopedic surgeons were asked what procedures they would commonly carry out in the scenario by selecting 1 or more of 12 options.

### Study Participants

The survey was limited to surgeons performing surgical procedures for foot deformities in CMT patients who were attending centers participating in the INC. Orthopedic surgeons were identified and contacted by investigators belonging to the INC and asked to complete the survey.

### Statistical Analysis

Median and range (minimum and maximum) were calculated for age of patients who underwent surgery. Frequency of foot deformities, use of orthotic aids, type of surgery, and number of patients who did not undergo surgery were expressed as a percentage. The chi‐square test was performed to compare patients with a diagnosis of CMT with other subtypes [hereditary sensory neuropathy (HSN), hereditary motor neuropathy (HMN), and hereditary neuropathy with liability to pressure palsies (HNPP)]. The non‐parametric Spearman test was performed to assess the correlation between number of interventions per year performed by individual surgeons and number of procedures suggested in response to clinical scenarios.

## RESULTS

### Patients' Details

Baseline data were obtained from 2,706 patients recruited between August 2009 and December 2014. Results are summarized in Table 1. There were 1,953 patients with a diagnosis of CMT, 72 with HMN, 65 with HSN, and 49 with HNPP. The precise diagnosis was not included for 567 patients entered in the natural history study. Patients' median age was 40 years, and ranged between 1 and 93 (interquartile range 19–55) years. Foot deformities were reported in 1,522 of 2,139 patients (71%) with a diagnosis of CMT and related inherited neuropathies (HNPP, HMN, and HSN) and were more common in patients with a diagnosis of CMT (1,443 of 1,953, 74%) (Table 1) compared with other subtypes (HMN, HSN, and HNPP; 79 of 186, 42%). Pes cavus and hammer toes were more commonly reported than pes planus in patients with CMT. Pes cavus was reported in 63% of patients with CMT1A. Shoe inserts and ankle‐foot orthoses were used, respectively, by 357 of 1,443 (25%) and 509 of 1,443 (35%) of CMT patients with foot deformities, by 13 of 39 (33%) and 16 of 39 (41%) of dHMN patients with foot deformities, by 8 of 24 (33%) and 7 of 24 (29%) of HSN patients with foot deformities, and by 2 of 16 (12%) and 4 of 16 (25%) of HNPP patients with foot deformities.

**Table 1:** Foot deformities and foot surgery in patients enrolled in the Natural History Study for CMT and related disorders

Diagnosis	*N*	Any foot deformity	Pes cavus	Hammer toes	Pes planus	Any foot surgery	Tendon transfer	Achilles tendon lengthening	Ankle joint fusion	Osteotomy^*^	Toes straightening	No surgery
CMT	1,953	1,443† (74%)	1,089 (56%)	534 (27%)	150 (8%)	420 (29%)	119 (10%)	108 (7%)	74 (5%)	100 (7%)	57 (5%)	1,023 (71%)
CMT1A	845	681† (80%)	532 (63%)	249 (29%)	56 (7%)	205 (30%)	54 (8%)	51 (7%)	37 (5%)	41 (6%)	23 (3%)	476 (70%)
CMTX	138	91 (66%)	72 (52%)	40 (29%)	7 (5%)	18 (20%)	4 (4%)	8 (9%)	1 (1%)	6 (6%)	2 (2%)	73 (83%)
HMN	72	39 (54%)	22 (30%)	11 (15%)	5 (7%)	12 (31%)	5 (10%)	6 (15%)	1 (2%)	2 (5%)	3 (8%)	27 (69%)
HSN	65	24 (37%)	8 (12%)	4 (6%)	9 (14%)	8 (33%)	2 (8%)	2 (8%)	2 (8%)	4 (16%)	3 (12%)	16 (66%)
HNPP	49	16 (37%)	9 (18%)	3 (6%)	4 (8%)	3 (18%)	2 (12%)	1 (6%)	1 (6%)	0	0	13 (81%)
Unspecified	567	392 (70%)	262 (46%)	142 (25%)	47 (8%)	131 (33%)	52 (13%)	33 (8%)	22 (6%)	20 (5%)	26 (7%)	261 (66%)
Total	2,706	1,914 (71%)	1,390 (51%)	694 (26%)	215 (8%)	574 (30%)	180 (9%)	150 (8%)	78 (5%)	126 (6%)	89 (5%)	1,318 (69%)

= ostetomy included calcaneal osteotomy, first metatarsal osteotomy, midfoot osteotomy.

=difference was statistically different (p < 0.0001) in CMT and CMT1A compared to other subtypes.

See text for abbreviations.

Overall, 30% of patients underwent foot surgery for correction of foot deformities; of these, 420 of 574 (73%) had a diagnosis of CMT and 205 of 420 (49%) had a diagnosis of CMT1A. Furthermore, 407 of 1,390 (29%) of the patients with pes cavus, 253 of 694 (36%) of the patients with hammer toes and 48 of 215 (22%) of the patients with pes planus had surgery. The ages of those who underwent surgery were available for 68% of the patients. Median age for any type of surgery was 15 (range 1‐73) years, with median ages for specific surgeries as follows: tendon transfer 16 (range 0.8–66) years; Achilles tendon lengthening 13 (range 0.8–73) years; osteotomy 17 (range 3–66) years; toe straightening 22 (range 9–66) years; and ankle arthrodesis 18 (range 8–64) years. Furthermore, 128 of 389 (33%) patients had surgery more than once and the median interval between first and last surgery was 5 (range 1–58) years. The surgical procedures performed are summarized in Table 1.

### Survey

Sixteen orthopedic surgeons working in selected INC CMT‐specialized centers worldwide were surveyed. Three surgeons were based in the UK, 2 in Italy, 3 in Australia, and 8 in the USA. Eight (50%) were pediatric orthopedic surgeons, 4 (25%) were adult foot and ankle orthopedic surgeons, 3 (19%) were both adult foot and ankle and pediatric orthopedic surgeons, and only 1 (6%) was a general orthopedic surgeon with an interest in foot and ankle and pediatric surgery. Five (31%) surgeons received > 15 referrals of patients with severe pes cavus per year and assessed >15 CMT patients per year, whereas the remainder received between 2 and 10 referrals per year. Six (37%) performed between 6 and 10 corrections per year for severe pes cavus.

**Figure 1:**
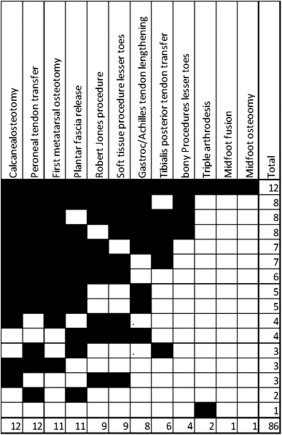
Paediatric clinical scenario Each column = surgical procedures; Each row represents the procedures chosen by each surgeon; Right hand column = total number of procedures suggested by each surgeon; bottom row = total number of times each procedure was chosen amongst surgeon responders.

**Figure 2:**
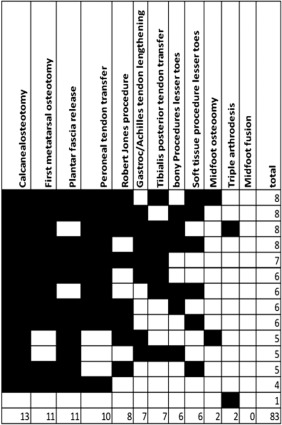
Adult clinical scenario. Each column = surgical procedures; Each row represents the procedures chosen by each surgeon; Right hand column = total number of procedures suggested by each surgeon; bottom row = total number of times each procedure was chosen amongst surgeon responders.

For both clinical scenarios, a variety of combinations of procedures commonly performed to correct pes cavus were selected by the surgeons. The median number of procedures per surgery was 5 (range 1–12) for the pediatric scenario and 6 (range 1–8) for the adult scenario. The most common procedures for the pediatric clinical scenario were calcaneal osteotomy and peroneal tendon transfer (both 12 of 16) and first metatarsal osteotomy and plantar fascia release (both 11 of 16) (Fig. 1). For the adult clinical scenario, 13 of 14 surgeons (93%) would commonly perform calcaneal osteotomy, 11 of 14 (78%) would commonly perform first metatarsal osteotomy or plantar fascia release, and 10 of 14 (71%) would perform peroneal tendon transfer (Fig. 2). Midfoot osteotomy, triple arthrodesis, and midfoot fusion were rarely selected in either scenario.

Overall, in both scenarios, the majority of surgeons (10 of 16) would perform at least calcaneal osteotomy and peroneal tendon transfer as part of the procedure to correct pes cavus. However, the exact combination varied considerably among surgeons, with only 2 surgeons selecting a similar combination of procedures to correct pes cavus on a pediatric case (calcaneal osteotomy, peroneal tendon transfer, first metatarsal osteotomy, and plantar fascia release) and none of the surgeons selecting an identical combination of procedures for an adult case. There was no correlation between numbers of interventions per year performed by individual surgeons and numbers of procedures suggested in response to clinical scenarios. There were no differences in terms of answers between surgeons from the USA and surgeons from the UK, Australia, and Italy.

## DISCUSSION

Our analysis of data collected from the large cohort of patients enrolled in an international natural history study showed that foot deformities were reported in 1,914 of 2,706 patients (71%) with CMT and related disorders, and 30% of patients required surgery for correction of foot deformities. Interestingly, 63% of patients with pes cavus had a diagnosis of CMT1A genetically confirmed, whereas pes cavus was less frequently observed in other subtypes of inherited neuropathy. These data confirm previous studies in which the presence of pes cavus ranged between 40% and 77% in CMT1A patients,^9^, ^10^, ^11^, ^12^ suggesting the role of muscular imbalance between anterolateral and posteromedial compartments in the pathogenesis of pes cavus.^12^ The relatively rarer presence of foot deformities in other subtypes of CMT, such as in HMN, could be explained by the differing muscular involvement with both anterior and posterior compartment muscles affected. These observations may be relevant for the management of orthopedic complications in CMT patients. Furthermore, in the present cohort, the use of orthoses (ankle‐foot orthosis and shoe inserts) was reported in 25%–41% of patients with CMT and related neuropathies and foot deformities. However, we did not study correlations between conservative measures and either need for orthopedic interventions or outcome of surgical interventions as these were outside the scope of this study.

Our survey, although limited by the small number of participating surgeons, highlights the variability of the surgical approach to management of orthopedic complications in CMT, even among centers with specific expertise in CMT. The cohort interviewed included both pediatric and adult surgeons who regularly operate on foot deformities in CMT patients. Interestingly, the approach to orthopedic management of patients with CMT varied considerably among surgeons, with only 2 surgeons agreeing on a similar combination of procedures in a pediatric case and none of the surgeons performing a similar combination of procedures in an adult case. These findings confirm the perception that surgical approach varies between different centers and between different countries. Therefore, both developing guidelines and identifying areas for research in surgery for CMT are very important.

It is noteworthy that the majority of the surgeons would perform at least calcaneal osteotomy and peroneal tendon transfer as part of procedures undertaken in both scenarios to correct pes cavus. The primary deformity in CMT is thought to be imbalance of the intrinsic foot muscles and/or an imbalance between tibialis anterior and peroneus longus giving rise to a plantarflexed first metatarsal and a pronated foot. The hindfoot varus is thought to be secondary—initially correctable, but becoming fixed with time. There was substantial disagreement on the role of metatarsal osteotomy in correcting deformity, with 11 of 14 surgeons performing metatarsal dorsiflexion osteotomy in the adult case and 9 of 16 performing the Robert Jones soft‐tissue procedure in the pediatric case. Controversy exists in the orthopedic literature as to whether the plantar fascia is tight in pes cavus. This was confirmed in our finding that 11 of 16 surgeons would perform a plantar fascia release in a pediatric patient and 11 of 14 surgeons would do a plantar fascia release in an adult patient. The use of various approaches among surgeons reflects a lack of understanding of the exact pathogenesis of foot deformities in CMT. It may be that the pathogenic mechanism is different in various types of CMT and further research is needed.

In conclusion, the results of this study on both the frequency of foot deformity in CMT and the approach to treatment, despite the limitations discussed, highlight the need for further research and the need for guidelines for foot surgery, which should include indications for and timing of surgery, type of surgery, and guidance for follow‐up. The development of guidelines will require careful follow‐up of patients who have had surgery to determine the long‐term outcomes for different procedures and need to take into consideration both medical and surgical perceived efficacy. We plan to undertake a workshop to develop a strategy for addressing these issues.

## Supporting information

Additional supporting information may be found in the online version of this article

Supporting Information
