# Supplementary material for: Prevalence and orthopedic management of foot and ankle deformities in Charcot–Marie–Tooth disease
Source: Muscle Nerve. 2017 Jul 7;57(2):255–9. doi: 10.1002/mus.25724 (PMC5811923; doi:10.1002/mus.25724)
Supplement: Supplementary file 1 — Supporting Information [file MUS-57-255-s001.pdf]

Orthopaedic Survey

How would you mostly describe your orthopaedic practice?

Adult foot ankle surgery only ☐

Paediatric orthopaedics only ☐

Both adult foot & ankle surgery and paediatric orthopaedics ☐

Lower limb surgery ☐

General orthopaedic surgery ☐

General orthopaedic surgery with an interest in foot and ankle surgery ☐

General orthopaedic surgery with an interest in paediatric orthopaedics ☐
General orthopaedic surgery with an interest in foot and ankle surgery and paediatric orthopaedics ☐

How many new referrals of patients with SEVERE pes cavus do you see per year?

<2 ☐

2-5 ☐

6-10 ☐

11-15 ☐

>15 ☐

How many patients with Charcot-Marie-tooth (HMSN) do you see per year?

<2 ☐

2-5 ☐

6-10 ☐

11-15☐

>15 ☐

How many corrections of the hindfoot in patients with SEVERE pes cavus do you perform per year (please do not include forefoot surgery)?

<2 ☐

2-5 ☐

6-10 ☐

11-15 ☐

>15 ☐

Consider a 13 year old child with Charcot-Marie-tooth with a typical pes cavus who has failed conservative management. What are the procedures that you would commonly carry out?

Gastrocnemius/Achilles lengthening ☐

Plantar fascia release ☐

Robert Jones Procedure ☐

Soft tissue procedure lesser toes ☐

Bony procedure lesser toes ☐

Calcaneal osteotomy ☐

First metalarsal osteotomy☐

Midfoot osteotomy ☐

Midfoot fusion +/- correction ☐

Triple arthrodesis ☐

Tibialis posterior tendon transfer ☐

Peroneal tendon surgery ☐

Consider a typical 24 year old patient with Charcot –Marie – Tooth, without any arthritic changes on radiographs and who has failed conservative treatment.
What are the procedures that you would commonly carry out?
(more than one response allowed)

Gastrocnemius/Achilles lengthening ☐

Plantar fascia release ☐

Robert Jones Procedure ☐

Soft tissue procedure lesser toes ☐

Bony procedure lesser toes ☐

Calcaneal osteotomy ☐

First metalarsal osteotomy ☐

Midfoot osteotomy ☐

Midfoot fusion +/- correction ☐

Triple arthrodesis ☐

Tibialis posterior tendon transfer ☐

Peroneal tendon surgery ☐
